# *In Vivo* Efficacy and Toxicity Studies of a Novel Antibacterial Agent: 14-*O*-[(2-Amino-1,3,4-thiadiazol-5-yl)Thioacetyl] Mutilin

**DOI:** 10.3390/molecules20045299

**Published:** 2015-03-24

**Authors:** Chao Zhang, Yunpeng Yi, Jiongran Chen, Rensheng Xin, Zhen Yang, Zhiting Guo, Jianping Liang, Ruofeng Shang

**Affiliations:** Key Laboratory of New Animal Drug Project of Gansu Province, Key Laboratory of Veterinary Pharmaceutical Development, Ministry of Agriculture, Lanzhou Institute of Husbandry and Pharmaceutical Sciences of CAAS, Lanzhou 730050, China; E-Mails: YJL.BZN@163.com (C.Z.); yiyp2015@163.com (Y.Y.); chenjiongran@126.com (J.C.); renshengxin0824@163.com (R.X.); yz19890502@126.com (Z.Y.); guozhiting197998@163.com (Z.G.)

**Keywords:** ATTM, ED_50_, acute toxicity, subchronic toxicity

## Abstract

A new pleuromutilin derivative with excellent antibacterial activity, 14-*O*-[(2-amino-1,3,4-thiadiazol-5-yl) thioacetyl] mutilin (ATTM), may serve as a possible lead compound for the development of antibacterial drugs. However, *in vivo* efficacy and toxicity evaluations of this compound have not been performed. In this study, we evaluated the efficacy of ATTM by measuring the survival of mice after a lethal challenge with methicillin-resistant *Staphylococcus aureus* (MRSA), and the 50% effective dose (ED_50_) was 5.74 mg/kg by the intravenous route. In an oral single-dose toxicity study, ATTM was orally administered to mice at different doses and the 50% lethal dose (LD_50_) was calculated to be 2304.4 mg/kg by the Bliss method. The results of the subchronic oral toxicity study in rats showed no mortality, exterior signs of toxicity, or differences in the total weight gain or relative organ weights between the treated groups and control group after administration. The hematological and serum biochemical data showed no differences between the treated and control groups, except for the levels of alkaline phosphatase (ALP), creatinine (CR) and blood glucose (GLU), which were significantly different in the high-dose group. The differences in the histopathological findings between the treated groups and the control group were not considered to be treatment-related. Our results indicated that the no observed adverse effect level (NOAEL) for ATTM was 5 mg/kg in this study.

## 1. Introduction

The increasing resistance of bacteria to the major classes of antibacterial drugs is becoming a serious threat to public health. Drug-resistant bacteria, especially methicillin-resistant *Staphylococcus aureus* (MRSA), currently cause infections in both hospitals and the worldwide community [1]. Therefore, new classes of antibacterial agents that work via novel mechanisms are urgently needed to effectively eradicate drug-resistant bacteria.

**Figure 1:**
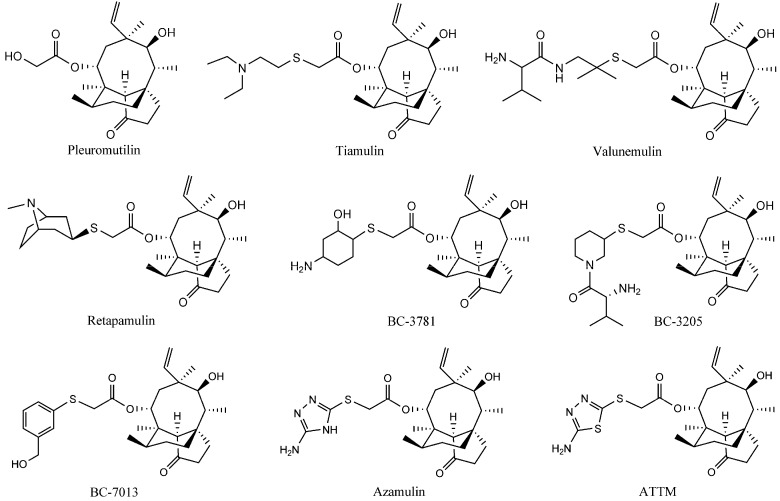
The structures of the pleuromutilin derivatives and ATTM.

The natural compound pleuromutilin (Figure 1) was first discovered and isolated from cultures of two species of basidiomycetes, *Pleurotusmutilus* and *P. passeckerianus*, in 1951 [2]. Modifications of pleuromutilin have led to three drugs: tiamulin, valnemulin, and retapamulin (Figure 1) [3,4]. Extensive efforts were made to synthesize three other compounds, BC-3781, BC-3205 and BC-7013 (Figure 1), for human use after the success of retapamulin [5,6]. Chemical footprinting studies showed that tiamulin and valnemulin bound to the bacterial ribosome at the peptidyl transferase center (PTC), thereby inhibiting the synthesis of the peptide bond by hindering the correct location of the amino acid on the tRNA [7,8]. Further studies demonstrated that the interactions of the tricyclic core of tiamulin are mediated through hydrophobic interactions and hydrogen bonds, which are formed mainly by the nucleotides of domain V [9,10].

A new pleuromutilin derivative, 14-*O*-[(2-amino-1,3,4-thiadiazol-5-yl) thioacetyl] mutilin (ATTM, Figure 1) is composed of a rather rigid 5-6-8 tricyclic carbon skeleton and a thiadiazole moiety [11]. This compound was structurally similar to azamulin (Figure 1), which was designed for human use during the early 1980s but did not undergo further clinical trials because of its poor solubility in water and its strong inhibition of human cytochrome P450s [12,13]. ATTM was first synthesized and evaluated in our lab. The *in vitro* antibacterial studies showed that it displayed excellent antibacterial activity against MRSA, methicillin-resistant *Staphylococcus epidermidis* (MRSE) and *Streptococcus agalactiae* (*S. agalactiae*). Preliminary pharmacokinetics studies in rats showed that ATTM may be able to serve as a possible lead compound for the development of antibacterial drugs [14]. This present report describes a subsequent pharmacological investigation of ATTM, including studies of the *in vivo* efficacy and acute toxicity in mice, and subchronic toxicity in rats.

## 2. Results and Discussion

### 2.1. In Vivo Efficacy of ATTM

A *staphylococcal* systemic infection model was used to evaluate the efficacy of ATTM by determining the survival of mice after a lethal challenge with MRSA. Tiamulin fumarate was chosen as the reference drug. After being infected with MRSA, the mice were intravenously treated with different doses of ATTM or tiamulin fumarate dissolved in vehicle. The survival rates of all groups are summarized in Table 1. Treatment with ATTM and tiamulin fumarate led to dose-dependent protection and the survival of the mice infected with MRSA, with a 50% effective dose (ED_50_) of 5.74 and 5.95 mg/kg body weight (b. w.); the confidence level of 95% was 3.75 to 8.78 mg/kg and 3.59 to 9.87 mg/kg, respectively). Thus, ATTM was more active than tiamulin fumarate against MRSA in this mouse systemic model.

**Table 1:** Survival of mice challenged with MRSA after treatment with different doses of ATTM.

Compounds	Group	*n*	Dose (mg/kg b. w.)	Logarithmic Dose	Survival	Survival Rate (%)
ATTM	1	10	2.5	0.4	2	20
	2	10	5.0	0.7	4	40
	3	10	10.0	1.0	9	90
	4	10	20.0	1.3	10	100
	5	10	40.0	1.6	10	100
Tiamulin fumarate	1	10	2.5	0.4	2	20
	2	10	5.0	0.7	4	40
	3	10	10.0	1.0	8	80
	4	10	20.0	1.3	10	100
	5	10	40.0	1.6	10	100

As previously reported [14], ATTM displayed excellent antibacterial activity *in vitro*, with MICs from 0.25–1 μg/mL against MRSA, MRSE and *S. agalactiae*. In the present study, the *in vivo* efficacy study showed that ATTM was able to protect animals in a dose-dependent fashion. These antibacterial studies have demonstrated that ATTM might act as a potent antibacterial drug.

### 2.2. Acute Oral Toxicity Study

The results of the oral single-dose toxicity study are summarized in Table 2. No animals died after receiving an oral dose of up to 948.15 mg/kg b. w. of ATTM. Conversely, all animals died when given the oral dose of 4800 mg/kg b. w. of ATTM. The approximate 50% lethal dose (LD_50_) in mice was determined to be 2304.4 mg/kg by the Bliss method, and the confidence level of 95% was 1861.4 mg/kg to 2870.5 mg/kg. No adverse effects or clinical signs of toxicity were observed for the surviving animals during the study. A necropsy of the surviving mice revealed no gross pathological finding and no significant differences in the liver, lungs, kidneys, heart, stomach, intestine, spleen or adrenal glands. However, drug precipitation was found in the stomachs of the mice that died during the early days after administration. Some areas of hemorrhage on the liver, lungs and small intestine were also found in some of mice that died during the treatment.

**Table 2:** Oral single-dose toxicity of ATTM in mice.

Group	*n*	Dose (mg/kg b. w.)	Logarithmic Dose	Mortality	Mortality Rate (%)
1	10	948.15	3.68	0	0
2	10	1422.22	3.51	2	20
3	10	2133.33	3.33	4	40
4	10	3200.00	3.15	7	70
5	10	4800.00	2.98	10	100
vehicle control group	10	20 (mL/kg b. w.)	-	0	0

The acute toxicity study of ATTM was conducted in mice to establish the potential for acute toxicity and to provide information pertaining to the upper dose limit that could be used in longer-term feeding studies. On the basis of the results, the estimated LD_50_ of ATTM to mice is 2304.4 mg/kg/b. w. Under the conditions of this study, a unique composition of ATTM and dimethyl sulfoxide (DMSO) did not produce any acute oral toxicological effects. This indicates that ATTM has relatively low toxicity and high potential for development as a new drug.

### 2.3. Subchronic Oral Toxicity

#### 2.3.1. Clinical Signs, Body Weights, and Food Consumption

After administration, no mortality or exterior signs of toxicity were observed in any of the dosing groups or the vehicle control group during the 28-day treatment period. The animals exhibited normal behavior and a normal physical condition, with no significant abnormalities in the clinical signs observed throughout the study.

**Figure 2:**
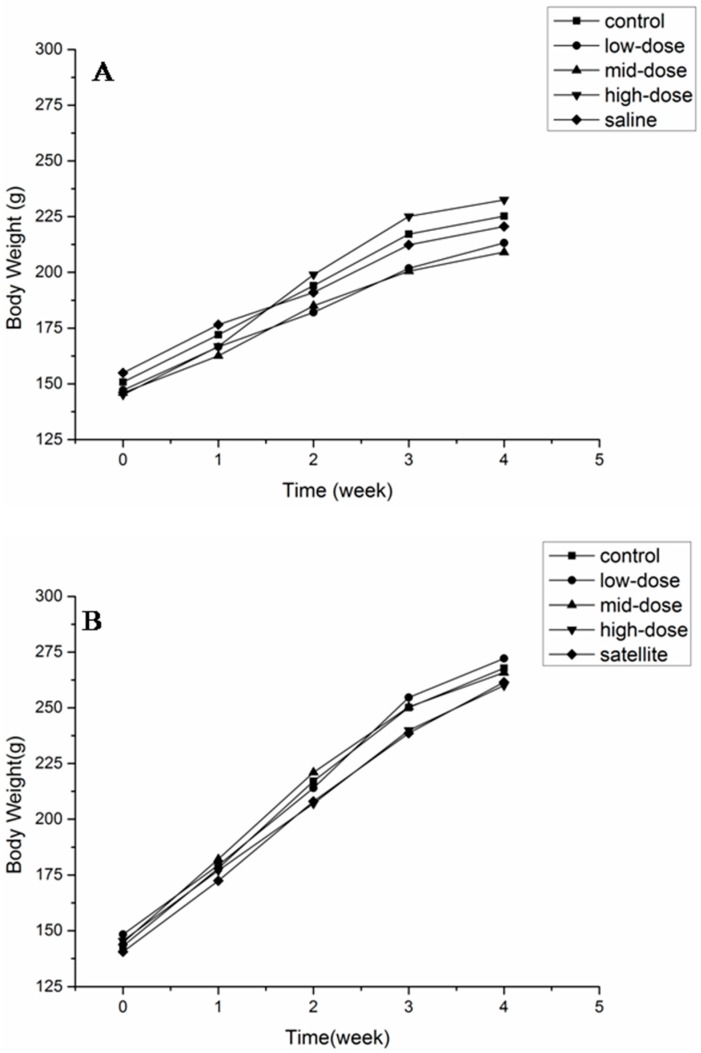
Body weight of female (**A**) and male (**B**) rats after a 28-day treatment with ATTM at 5, 25 and 125 mg/kg b. w. per day (*n* = 10).

The body weights of animals of both sexes are shown in Figure 2. No significant differences were observed in any of the groups at any time point. The body weight and food consumption are shown in Table 3. There was no difference in the total weight gain and the average daily food intake between the treated and control groups.

**Table 3:** Body weight gain and food consumption of rats orally administered ATTM for 28 days.

Item	Control Group	ATTM-Treated Groups (mg/kg b. w./d)			Saline Group
		Low-Dose (5)	Middle-Dose (25)	High-Dose (125)	
Females					
Total body weight gain (g)	74.4 ± 7.5	66.06 ± 5.8	63.07 ± 6.3	87.4 ± 4.9	65.7 ± 8.5
Daily food consumption (g)	118.6 ± 7.2	107.1 ± 13.6	106.9 ± 10.8	106.8 ± 15.7	109.7 ± 9.9
Males					
Total body weight gain (g)	124.8 ± 10.4	123.8 ± 9.6	121.1 ± 8.2	114.5 ± 9.9	121.0 ± 5.6
Daily food consumption (g)	148.4 ± 10.2	134.9 ± 7.5	147.7 ± 12.6	147.7 ± 12.6	157.1 ± 8.6

The values expressed as mean ± SD (*n* = 10/sex/dose).

#### 2.3.2. Hematological and Serum Biochemical Data

The hematological and serum biochemical data on the examination on day 29 are summarized in Table 4 and Table 5. There were no significant differences of the hematological data between the treated groups and control group. The level of alkaline phosphatase (ALP) was significantly decreased in the female high-dose group (*p* < 0.01). The creatinine (CR) and blood glucose (GLU) levels were significantly increased in both the female and male high-dose groups (*p* < 0.05).

Blood biochemical determinations that investigate major toxic effects on specific tissues, specifically the kidneys and liver, may provide useful information about the mechanism of toxicity of the agent. Some enzymes and proteins can indicate the impact on the renal function (such as CR) [15] or liver (such as ALP and GLU) [16]. Significant differences in the CR, ALP and GLU levels were found in the treated groups compared with the control group. These results suggest that ATTM may alter both the renal and hepatic function, indicating that the rats’ kidneys and livers in the high-dose group were injured after the administration of 125 mg/kg b. w. of ATTM.

**Table 4:** Hematological findings in rats orally administered ATTM for 28 days.

Hematological Parameters	Control group	ATTM-Treated Groups (mg/kg b. w./d)			Saline Group
		Low-Dose (5)	Middle-Dose (25)	High-Dose (125)	
Females					
WBC(10^9^/L)	14.2 ± 4.5	10.9 ± 3.6	9.3 ± 7.5	10.2 ± 2.5	15.2 ± 3.8
RBC (10^12^/L)	8.33 ± 0.78	7.47 ± 0.65	7.85 ± 0.48	7.7 ± 0.54	8.9 ± 0.74
HGB (g/L)	180 ± 7.4	179 ± 6.3	171 ± 5.2	170 ± 7.8	180 ± 7.1
HCT%	0.504 ± 1.4	0.392 ± 1.7	0.464 ± 1.9	0.414 ± 1.6	0.519 ± 1.4
MCV (fL)	60.5 ± 0.89	60.6 ± 0.92	59.1 ± 0.95	61.8 ± 0.91	58.3 ± 0.93
MCH (pg)	21.6 ± 0.90	21.5 ± 0.88	21.8 ± 0.87	22.4 ± 0.79	21.6 ± 0.92
MCHC (g/L)	357 ± 0.52	355 ± 0.48	369 ± 0.58	362 ± 0.41	347 ± 0.61
PLT (10^9^/L)	1211 ± 127.83	1154 ± 153.47	1137 ± 184.26	1224 ± 192.38	1237 ± 83.54
Males					
WBC (10^9^/L)	10.6 ± 2.8	10.9 ± 2.3	12.5 ± 4.6	12.2 ± 3.5	13.3 ± 3.9
RBC (10^12^/L)	8.37 ± 0.64	8.21 ± 0.61	8.64 ± 0.69	8.7 ± 0.66	8.18 ± 0.65
HGB (g/L)	182 ± 5.4	172 ± 5.6	189 ± 5.9	178 ± 6.3	179 ± 7.1
HCT%	0. 520 ± 1.3	0.461 ± 1.6	0.535 ± 1.9	0.474 ± 1.2	0.485 ± 1.7
MCV (fL)	62.1 ± 0.75	56.2 ± 0.72	61.9 ± 0.79	62.8 ± 0.64	59.3 ± 0.68
MCH (pg)	21.7 ± 0.83	21.0 ± 0.95	21.9 ± 0.78	22.4 ± 0.74	20.7 ± 0.87
MCHC (g/L)	350 ± 0.56	373 ± 0.62	353 ± 0.64	362 ± 0.58	348 ± 0.64
PLT (10^9^/L)	1152 ± 118.54	1143 ± 178.56	1229 ± 139.72	1224 ± 157.56	1215 ± 137.83

The values expressed as mean ± SD (*n* = 10/sex/dose).

**Table 5:** Blood biochemical parameters of rats orally administered ATTM for 28 days.

Biochemical Parameters	Control Group	ATTM-Treated Groups (mg/kg b. w./d)			Saline Group
		Low-Dose (5)	Middle-Dose (25)	High-Dose (125)	
Females					
ALT (U/L)	52 ± 5.2	48 ± 4.6	45 ± 5.8	44 ± 6.1	64 ± 6.5
ALP (U/L)	266 ± 14.5	233 ± 13.6	251 ± 16.8	153 ± 19.2 **	278 ± 18.4
AST (U/L)	110 ± 23.7	113 ± 27.5	112 ± 26.5	106 ± 23.4	112 ± 24.6
T-Bil (μmol/L)	4.70 ± 0.36	4.3 ± 0.54	4.83 ± 0.82	3.71 ± 0.71	3.68 ± 0.59
LDH (U/L)	761 ± 12.8	770 ± 11.6	785 ± 13.2	797 ± 14.1	721 ± 13.7
TC (mmol/L)	2.6 ± 0.21	2.6 ± 0.17	2.3 ± 0.19	2.7 ± 0.20	1.9 ± 0.45
HDL-c (mmol/L)	1.83 ± 0.14	1.86 ± 0.17	1.70 ± 0.12	2.03 ± 0.16	1.80 ± 0.19
LDL-c (mmol/L)	0.47 ± 0.07	0.43 ± 0.05	0.36 ± 0.08	0.35 ± 0.04	0.32 ± 0.09
TG (mmol/L)	0.68 ± 0.31	0.60 ± 0.45	0.74 ± 0.41	0.76 ± 0.62	0.80 ± 0.58
CK (U/L)	2178 ± 33.6	2127 ± 37.6	2160 ± 41.4	2007 ± 32.9	1983 ± 36.4
CR (μmol/L)	66 ± 4.7	66 ± 4.6	65 ± 5.3	73 ± 6.2 *	68 ± 4.5
Urea (mmol/L)	5.4 ± 5.6	6.0 ± 7.8	8.8 ± 8.1	6.1 ± 6.5	5.7 ± 6.2
UA (μmol/L)	79.5 ± 0.24	82.6 ± 0.37	80.2 ± 0.19	80.2 ± 0.56	83.9 ± 0.47
TP (g/L)	60.3 ± 4.7	57.9 ± 3.8	63.6 ± 5.3	65.6 ± 4.2	69.1 ± 4.9
ALB (g/L)	30.2 ± 2.4	22.4 ± 2.7	33.6 ± 3.2	31.4 ± 3.8	30.8 ± 3.0
GLU (mmol/L)	4.24 ± 0.35	3.94 ± 0.37	5.09 ± 0.32	6.7 ± 0.39 *	5.11 ± 0.31
Ca (mmol/L)	2.70 ± 0.10	2.90 ± 0.08	2.49 ± 0.12	2.41 ± 0.14	2.44 ± 0.13
P (mmol/L)	2.7 ± 0.24	2.7 ± 0.33	3.9 ± 0.45	3.3 ± 0.38	2.4 ± 0.51
Males					
ALT (U/L)	41 ± 5.4	49 ± 6.4	48 ± 6.1	44 ± 4.8	47 ± 3.9
ALP (U/L)	103 ± 14.7	116 ± 15.4	110 ± 17.4	113 ± 18.9	117 ± 16.8
AST (U/L)	117 ± 23.5	112 ± 25.6	110 ± 21.9	106 ± 26.5	116 ± 27.8
T-Bil (μmol/L)	5.12 ± 0.34	3.44 ± 0.37	4.49 ± 0.36	3.71 ± 0.45	4.8 ± 0.52
LDH (U/L)	518 ± 12.3	512 ± 12.7	515 ± 11.8	517 ± 13.6	517 ± 14.2
TC (mmol/L)	2.3 ± 0.25	2.3 ± 0.29	2.2 ± 0.21	2.7 ± 0.19	3.2 ± 0.35
HDL-c (mmol/L)	1.77 ± 0.15	1.73 ± 0.16	1.59 ± 0.13	2.03 ± 0.17	2.24 ± 0.15
LDL-c (mmol/L)	0.29 ± 0.08	0.35 ± 0.06	0.27 ± 0.04	0.35 ± 0.05	0.52 ± 0.10
TG (mmol/L)	0.65 ± 0.36	0.69 ± 0.39	0.56 ± 0.32	0.76 ± 0.47	1.17 ± 0.35
CK (U/L)	2208 ± 42.5	2178 ± 32.6	2116 ± 35.7	2207 ± 36.2	2287 ± 37.9
CR (μmol/L)	69 ± 4.7	67 ± 4.5	68 ± 5.2	73 ± 6.7 *	68 ± 5.8
Urea (mmol/L)	6.6 ± 6.5	6.1 ± 7.2	6.2 ± 5.8	6.1 ± 4.9	6.3 ± 8.1
UA (μmol/L)	89.4 ± 0.51	64.5 ± 0.27	82.9 ± 0.46	80.2 ± 0.35	84.0 ± 0.49
TP (g/L)	68.7 ± 4.7	60.5 ± 5.6	66.8 ± 3.9	65.6 ± 6.1	64.1 ± 5.8
ALB (g/L)	32.3 ± 2.5	29.5 ± 3.9	32.5 ± 3.2	31.4 ± 4.5	34.9 ± 3.7
GLU (mmol/L)	5.20 ± 3.2	5.22 ± 3.8	5.29 ± 3.3	5.70 ± 3.5 *	5.11 ± 3.1
Ca (mmol/L)	2.64 ± 0.13	2.44 ± 0.17	2.39 ± 0.09	2.41 ± 0.13	2.60 ± 0.11
P (mmol/L)	3.0 ± 0.36	2.8 ± 0.29	2.5 ± 0.37	2.4 ± 0.42	2.6 ± 0.24

The values expressed as mean ± SD (*n* = 10/sex/dose). * *p* < 0.05 as compared with control group; ** *p* < 0.01 as compared with control group.

#### 2.3.3. Organ Weights

The relative organs weights of male and female rats treated with ATTM orally for 28 days are summarized in Table 6. The relative liver weight was significantly decreased in the high-dose groups in both females and males. No significant differences were observed for other organs in either gender.

**Table 6:** The relative organ weight of rats orally administered ATTM for 28 days.

Item	Control Group	ATTM-Treated Groups (mg/kg b. w./d)			Saline Group
		Low-Dose (5)	Middle-Dose (25)	High-Dose (125)	
Females					
Heart	0.40 ± 0.12	0.39 ± 0.28	0.39 ± 0.26	0.40 ± 0.18	0.37 ± 0.29
Liver	3.06 ± 0.26	3.49 ± 0.35	3.52 ± 0.38	4.01 ± 0.25 *	3.30 ± 0.17
Spleen	0.28 ± 0.05	0.23 ± 0.04	0.24 ± 0.03	0.27 ± 0.02	0.26 ± 0.06
Lung	0.60 ± 0.15	0.75 ± 0.13	0.62 ± 0.17	0.61 ± 0.19	0.66 ± 0.21
Kidney	0.74 ± 0.13	0.73 ± 0.17	0.80 ± 0.14	0.80 ± 0.18	0.66 ± 0.17
Thymus	0.20 ± 0.09	0.20 ± 0.06	0.16 ± 0.04	0.20 ± 0.05	0.21 ± 0.08
Ovaries	0.06 ± 0.01	0.07 ± 0.03	0.06 ± 0.04	0.06 ± 0.07	0.04 ± 0.05
Males					
Heart	0.37 ± 0.14	0.34 ± 0.18	0.35 ± 0.26	0.34 ± 0.23	0.31 ± 0.27
Liver	3.14 ± 0.25	3.16 ± 0.28	2.99 ± 0.24	3.98 ± 0.27 *	3.03 ± 0.19
Spleen	0.22 ± 0.06	0.17 ± 0.07	0.22 ± 0.03	0.20 ± 0.02	0.27 ± 0.08
Lung	0.57 ± 0.14	0.63 ± 0.13	0.56 ± 0.18	0.60 ± 0.20	0.66 ± 0.25
Kidney	0.86 ± 0.12	0.78 ± 0.15	0.83 ± 0.18	0.80 ± 0.21	0.76 ± 0.19
Thymus	0.15 ± 0.04	0.10 ± 0.07	0.29 ± 0.11	0.19 ± 0.09	0.17 ± 0.12
Testes	1.16 ± 0.12	1.33 ± 0.18	1.27 ± 0.24	1.30 ± 0.32	1.21 ± 0.25

The values expressed as mean ± SD (*n* = 10/sex/dose). The relative organ body weight ratio $=\frac{Absolute\;organ\;weight\;(g)}{Body\;weight\;of\;rats\;on\;sacrifice\;day\;(g)}\times 100$. * *p* < 0.05 as compared with control group.

#### 2.3.4. Histopathology Examination

**Figure 3:**
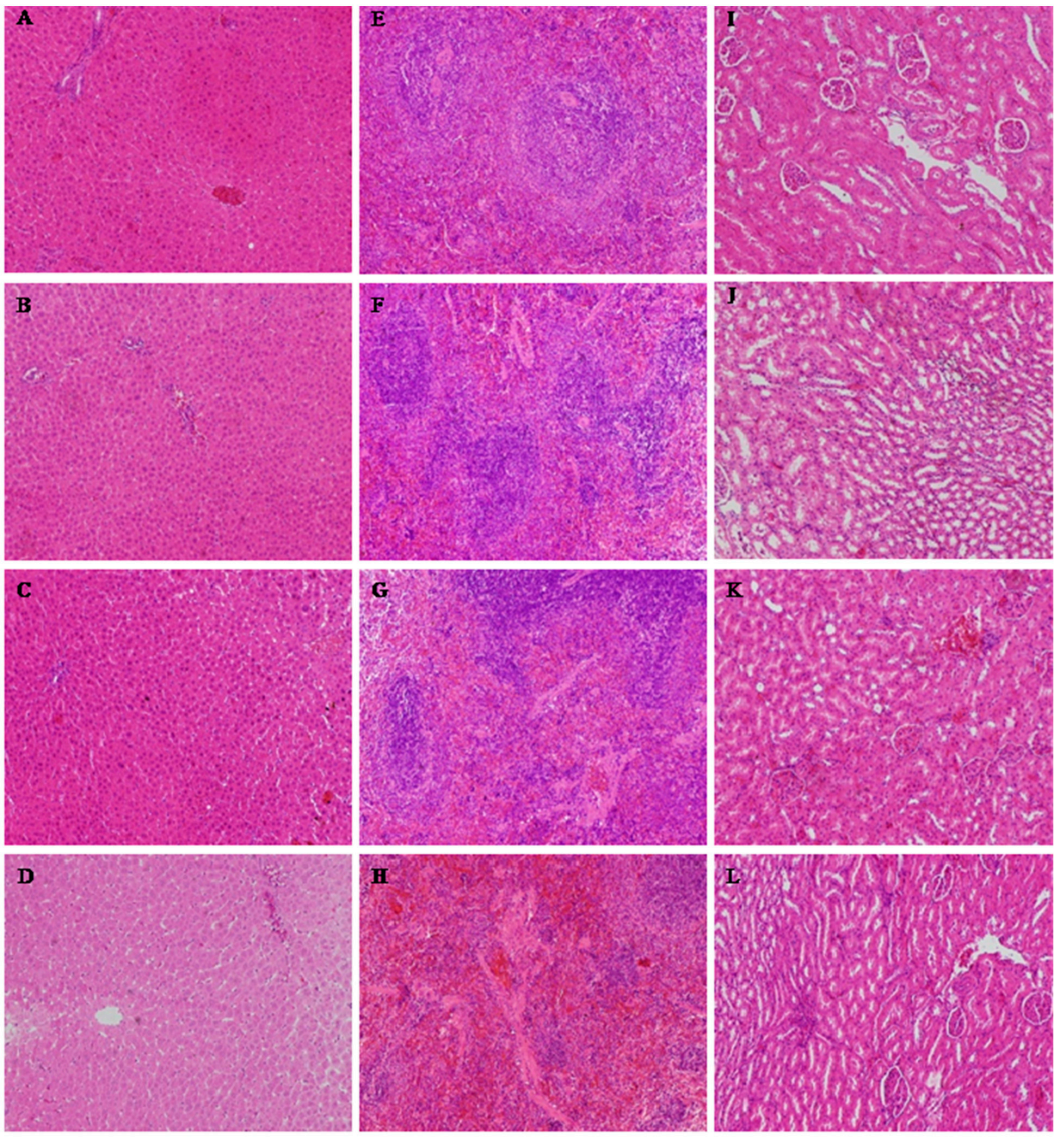
The results of the histopathological examinations of rats orally administered ATTM for 28 days (H&E × 100). (**A**–**D**) liver; (**E**–**H**) spleen; (**I**–**L**) kidney. (**A**, **E** and **I**) control group; (**B**, **F** and **J**) low-dose group; (**C**, **G** and **K**) middle-dose group; (**D**, **H** and **L**) high-dose group.

The histological alterations of the heart, liver, spleen, lungs, kidneys, thymus, ovaries or testes were observed after 28 days. Histopathological examinations of the heart, lungs, thymus, ovaries or testes showed no abnormalities in any of the treatment groups (data not shown). The histopathological examination of the liver, spleen and kidneys (Figure 3) showed that there was occasionally slight degeneration and necrosis in the liver, which was most obvious in the high-dose group. The spleens of the animals in the middle-dose, low-dose and control groups were normal, but there was splenic corpuscle atrophy and an unclear boundary between the red and white pulp in the high-dose group. The kidneys of the middle-dose group presented mild renal tubular cell necrosis and degeneration, but these were most serious in the high-dose group. The kidneys of the low-dose group and control animals were normal.

A histopathological examination of the liver, spleen and kidneys in the high-dose group showed some toxicological effects. However, no significant changes in the low-dose ATTM group were found in the hematological and blood biochemical analysis compared to the control group, which was in agreement with the histopathological finding. These results indicate that ATTM fed at 5 mg/kg for 28 days is generally considered to be safe for rats. We inferred that the target organs are the liver, spleen and kidneys, and the no observed adverse effect level (NOAEL) was 5 mg/kg in this study.

## 3. Experimental Section

### 3.1. Preparation of ATTM

ATTM was synthesized in our lab, as previously reported [14]. The synthesis of ATTM begins from 22-*O*-tosylpleuromutilin, which was obtained by the reaction of pleuromutilin and p-toluenesulfonyl chloride to activate the 22-hydroxyl of pleuromutilin. The intermediate, 14-*O*-(iodoacetyl)-mutilin was prepared in good yield under reflux for 3 h in acetone. Finally, ATTM was obtained in 82% yield by the nucleophilic attack of 2-amino-5-mercapto-1,3,4-thiadiazole on 14-*O*-(iodoacetyl)-mutilin under alkaline conditions.

### 3.2. Animals

Adult specific pathogen free (SPF) Kunming mice (weighing 18–22 g) and Sprague-Dawley (SD) rats (aged 3–4 weeks, weighing 140–160 g) were purchased from the Laboratory Animal Center of Lanzhou University (Lanzhou, China) and maintained under controlled temperature conditions (23 °C), with a constant 12 h light-dark cycle and free access to food and water. The experimental procedures were performed in accordance with the Ethical Principles in Animal Research and were approved by the Committee for Ethics in the Laboratory Animal Center of Lanzhou University (number: SCXK2013-0002).

### 3.3. In Vivo Efficacy in a Mouse Model

Male and female mice were rendered neutropenic upon treatment for four days with 150 mg/kg cyclophosphamide intraperitoneally followed by 100 mg/kg for one day, after they had been acclimated for five days. The neutropenic mice (10 per group) received a 0.5 mL MRSA inoculum of 10^9^ CFU/mL via intraperitoneal (ip) injection. At one hour post infection, the mice were then intravenously (iv) administered ATTM dissolved in 0.5 mL vehicle (soybean lecithin: sterile water = 1:30) at doses of 2.5, 5, 10 and 20 mg/kg b. w. Tiamulin fumarate was used as a control in the same manner and at the same doses as ATTM. The survival of mice at 72 h after infection was used as the end point and the ED_50_ was calculated by the method described by Reed Muench [17] using the Hill equation.

### 3.4. Acute Oral Toxicity in Mice

Thirty male and thirty female SPF Kunming mice were stratified by weight and randomly assigned to six groups: five treatment groups and one vehicle control group, with five male and five female mice for each group. The vehicle control group received DMSO in a volume of 20 mL/kg b. w. by oral gavage. The ATTM was dissolved in DMSO and administered to the mice at doses of 948.15, 1422.22, 2133.33, 3200.00 and 4800.00 mg/kg body weigh, respectively, and all animals were observed twice daily for symptoms and mortality for one week. The vehicle control group was observed at the same time. All of the surviving animals were euthanized at the end of the study, and their vital organs were individually observed for overt pathology by necropsy, and the LD_50_ was calculated by the Bliss method on day 8.

### 3.5. Subchronic Oral Toxicity

#### 3.5.1. Experimental Design

A total of 100 healthy male and female SD rats (140–160 g) were selected and randomly divided into five groups (20 rats in each group, male: female = 1:1) after one week of acclimatization. One group of rats was assigned to serve as control group, and these animals received DMSO in a volume of 4 mL/kg b. w., and another group was assigned to a saline group and received physiological saline in the same volume as the DMSO by oral gavage. The other three groups were assigned to ATTM treatment groups, and received ATTM via oral gavage at doses of 5, 25 and 125 mg/kg b. w. per day (at the same time each day ± 1 h). The animals in all groups were used for 28-day subchronic toxicity study, and were observed once daily to detect signs of toxicity. The animals’ body weight and food consumption were measured every week during the whole observation period, and the gavage volume was adjusted based on the last measured body weight.

#### 3.5.2. Clinical Signs, Body Weights and Food Consumption

The rats were observed for behavioral changes, mortality, and symptoms and signs of gross toxicity at least once per day during the 28-day subchronic toxicity study. The individual body weights of the rats were measured and recorded at least once a week. The mean weekly body weight gain was calculated for each sex and dose level during the entire test period. Individual food consumption was measured and recorded weekly. The mean daily food consumption was calculated for each sex and dose level for each weekly interval and for the overall test period.

#### 3.5.3. Blood Analysis

At the end of the experiment, blood was collected from the femoral artery in ethylenediaminetetraacetic acid (EDTA) coated tubes for hematology studies and in non-oxalate tubes for the separation of serum on the 29th day following a 12-h fast. The hematological analyses included the white blood cell (WBC), red blood cell (RBC) counts, the hemoglobin (HGB) and hematocrit (HCT) levels, mean corpuscular volume (MCV), mean corpuscular hemoglobin (MCH), mean corpuscular haemoglobin concentration (MCHC) and number of platelets (PLT), which were determined using a Poche-100iv Diff instrument (SYSMEX, Kakogawa, Japan).

The other blood collected in non-oxalate tubes was centrifuged at 3500 rpm (15 min at 4 °C) and the supernatant (serum) was collected and introduced into new tubes for the subsequent biochemical analyses of the levels of alanine transaminase (ALT), ALP, aspartate transaminase (AST), total bilirubin (T-Bil), lactate dehydrogenase (LDH), total cholesterol (TC), high density lipoprotein (HDL), low density lipoprotein (LDL), triglycerides (TG), creatine kinase (CK), CR, urea nitrogen (Urea), uric acid (UA), total protein (TP), albumin (ALB), GLU, Ca and P. These were measured using reagent kits and a Mindray BS-420 auto hematology analyzer (Mindray Corporation, Shenzhen, China).

#### 3.5.4. Necropsy, Organ Weight and Histopathology

Finally, the rats were anesthetized using an excess of CO_2_ anesthesia, followed by exsanguinations. The gross pathological changes were recorded for each rat, and the heart, liver, kidneys, spleen, thymus, lungs, testes and ovaries were collected, weighed and the relative weights (organ weight (g)/100 g b. w.) were recorded. Histopathological examinations were performed on the heart, liver, kidneys, spleen, thymus, lungs, testes or ovaries and small intestines of each rat.

### 3.6. Statistical Analysis

The data were expressed as the mean ± standard deviation (SD) and were analyzed using the SAS statistical software package (Version 9.0; SAS Institute, Cary, NC, USA) to account for the effects of ATTM on the weight, hematological findings and organ effects. A parametric one-way analysis of variance (ANOVA) for repeated measures was used to examine intergroup differences. When a significant difference (*p* < 0.05) was found, Tukey’s test was used to compare the means.

## 4. Conclusions

Historically, the semi-synthesis of new compounds based on natural products, especially complex natural products, has been the predominant avenue to the development of new antibiotics. The chemical modifications of pleuromutilin were made in an attempt to improve the antimicrobial activity and *in vivo* efficacy of the compounds after the identification of the structure of pleuromutilin. ATTM, a new derivative of pleuromutilin bearing a thiadiazole moiety, was synthesized in our lab. The *in vivo* efficacy study showed that ATTM exhibited potent activity, with an ED_50_ of 5.74 mg/kg b. w. when used to treat MRSA infected mice. Moreover, a study in mice showed no evidence of acute toxicity after the administration of an oral dose of up to 948.15 mg/kg b. w. of ATTM, with an approximate LD_50_ of 2304.4 mg/kg determined by the Bliss method. No animals died and no clinical abnormalities were observed that were considered to be associated with the 28-day treatment. Most of the hematological and serum biochemical data showed no differences between the treated groups and control group. Some toxic effects could be found in the liver, spleen and kidneys in the high-dose group by the histopathological examination, but these were not considered treatment-related. Our results show that ATTM fed at 5 mg/kg for 28 days is generally considered to be safe for rats.

The above results show that ATTM has a relatively high efficacy *in vivo* and has a low toxicity profile as a new candidate drug. However, other laboratory studies based on protocols by regulatory agencies should be performed to evaluate the drug potential of this compound.
